# A poxvirus Bcl-2-like gene family involved in regulation of host immune response: sequence similarity and evolutionary history

**DOI:** 10.1186/1743-422X-7-59

**Published:** 2010-03-15

**Authors:** José M González, Mariano Esteban

**Affiliations:** 1 Department of Molecular and Cellular Biology, Centro Nacional de Biotecnología - CSIC, Darwin 3, 28049 Madrid, Spain

## Abstract

**Background:**

Poxviruses evade the immune system of the host through the action of viral encoded inhibitors that block various signalling pathways. The exact number of viral inhibitors is not yet known. Several members of the vaccinia virus A46 and N1 families, with a Bcl-2-like structure, are involved in the regulation of the host innate immune response where they act non-redundantly at different levels of the Toll-like receptor signalling pathway. N1 also maintains an anti-apoptotic effect by acting similarly to cellular Bcl-2 proteins. Whether there are related families that could have similar functions is the main subject of this investigation.

**Results:**

We describe the sequence similarity existing among poxvirus A46, N1, N2 and C1 protein families, which share a common domain of approximately 110-140 amino acids at their C-termini that spans the entire N1 sequence. Secondary structure and fold recognition predictions suggest that this domain presents an all-alpha-helical fold compatible with the Bcl-2-like structures of vaccinia virus proteins N1, A52, B15 and K7. We propose that these protein families should be merged into a single one. We describe the phylogenetic distribution of this family and reconstruct its evolutionary history, which indicates an extensive gene gain in ancestral viruses and a further stabilization of its gene content.

**Conclusions:**

Based on the sequence/structure similarity, we propose that other members with unknown function, like vaccinia virus N2, C1, C6 and C16/B22, might have a similar role in the suppression of host immune response as A46, A52, B15 and K7, by antagonizing at different levels with the TLR signalling pathways.

## Background

Innate immune cells recognize pathogens through pattern-recognition receptors (PRRs) [1]. PRRs include Toll-like receptors (TLRs), RIG-I-like receptors and NOD-like receptors. Pathogen recognition activates an immune response through signalling pathways that trigger the expression of genes encoding Type I IFNs and pro-inflammatory cytokines. Poxvirus genomes contain a large number of genes involved in avoiding the host immune response to viral infection [2,3]. Known examples are vaccinia virus (VACV) genes coding for proteins A46, A52, B15, K7 and N1, which interfere with TLR signalling pathway at different levels. A46 contains a putative Toll/Interleukin-1 receptor (TIR) domain and targets several TIR adaptors like MyD88, MAL (TIRAP), TRIF and TRAM [4,5], thus blocking MAP kinase activation and TRIF-mediated IRF3 activation. A52 targets IRAK2 and TRAF6, and has a greater effect than A46 on inhibiting the activation of NF-kappaB [4,6]. Strikingly, it has been reported that A52 also activates p38 MAPK and potentiates LPS-induced IL-10 [7]. Sequence relationship between A52 and N1 proteins led to experiments that related N1 with the inhibition of NF-kappaB activation by several signalling pathways [8]. N1 is an intracellular homodimer that has been shown to associate with several components of the IKK complex and with TANK-binding kinase 1 (TBK1) thus inhibiting NF-kappaB and IRF3 activation, respectively [8,9], although recent experiments could not reproduce these interactions [10,11]. The crystallographic structure of N1 reveals a surprising similarity to Bcl-2 family of apoptotic regulators despite the absence of sequence homology [11,12]. Moreover N1 binds with high affinity to BH3 peptides from pro-apoptotic proteins Bid, Bim and Bak [12] and even inhibits the increase in mitochondrial membrane permeability and caspase 3/7 activation after apoptotic stimuli [11]. B15 (named B14 in VACV strain Western Reserve) is an intracellular virulence factor [13], and has been found to target the IKK complex by avoiding IKKbeta phosphorylation and subsequent IKK activation which would lead to degradation of IkappaB, the inhibitor of NF-kappaB [10]. The crystallographic structures of A52 and B15 have been recently solved, showing that both are homodimers with a Bcl-2-like fold similar to that of N1 [14]. But in contrast to N1 the BH3-peptide-binding groove in both structures is occluded, what may explain why they cannot protect staurosporine-treated cells from apoptosis [14]. Similarly to A52, K7 inhibits TLR-induced NF-kappaB activation and interacts with IRAK2 and TRAF6 [15]. Besides, K7 has been shown to modulate innate immune signalling pathways by binding the cellular DEAD-box RNA helicase DDX3, which forms part of a complex with TBK1-IKKepsilon that activates IRF3, thus inhibiting the IRF3-mediated IFNbeta gene transcription. This interaction was not observed in the case of A52. A NMR solution structure of K7 reveals a monomer that adopts a Bcl-2 fold, although similarly to A52 and B15 its pro-apoptotic peptide binding groove is predicted not to be functional [16]. The molecular details of the K7-DDX3 interaction have recently been unveiled [17].

In the Pfam database of protein families and domains [18] A46, A52, B15 and K7 are included in a single family (Pox_A46) together with other poxvirus proteins like VACV C6 and C16/B22, whereas N1 is classified in the Orthopox_N1 family. Because of the importance of host immune response modulation for poxviruses we hypothesized the existence of additional genes involved in this role among those of still unknown function. Hence, in this investigation we have searched for homologues of Pox_A46 family within poxvirus genomes using bioinformatics tools. We have found a clear relationship of A46 family not only with N1 but also with poxvirus N2 and C1 protein families, suggesting that these proteins probably adopt a common structural fold. The sequence relationship existing among these four families is presented. These similarities indicate that VACV C6, C16/B22, N2 and C1, whose function is currently unknown, may be involved in suppressing the host immune response through the inhibition of either apoptosis or the TLR signalling pathway. In addition we show that this family is present exclusively in a monophyletic subset of vertebrate poxviruses. The reconstruction of the evolutionary history of this gene family indicates numerous gene gain events in more remote ancestral genomes and a further stabilization of the gene contents in extant genomes.

## Results and Discussion

### Poxvirus A46, N1, N2 and C1 protein families share a common domain

**Figure 1:**
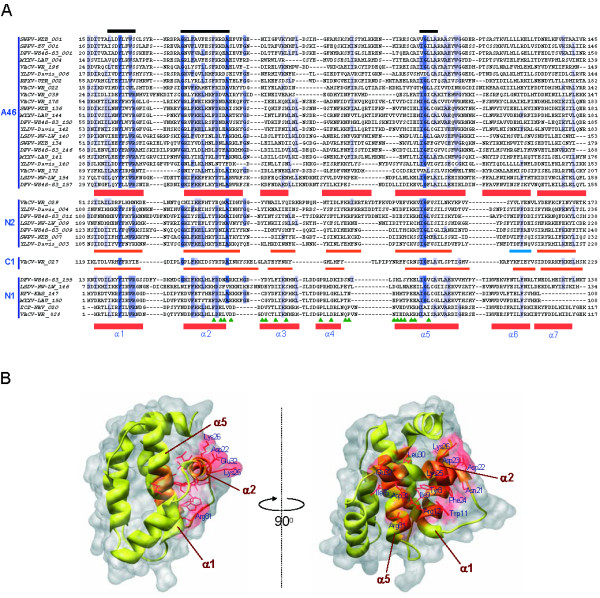
**Sequence conservation in A46 and related families**. (A) Multiple sequence alignment with the common sequence domain found in protein families A46, N1, N2 and C1. The alignment is non-redundant at 90% sequence identity. Sequences are identified by species/strain and gene locus number: SWPV-NEB, swinepox virus strain Nebraska 17077-99; SPPV-TU, sheeppox virus strain TU-V02127; DPV-W848_83, deerpox virus strain W-848-83; MYXV-LAU, myxoma virus strain Lausanne; RFV-KAS, rabbit fibroma virus strain Kasza; VACV-WR, vaccinia virus strain Western Reserve; YLDV-Davis, yaba-like disease virus strain Davis; RPXV-UTR, rabbitpox virus strain Utrecht; LSDV-NW_LW, lumpy skin disease virus strain Neethling Warmbaths LW; ECTV-NAV, ectromelia virus strain Naval. Shading indicates degree of sequence similarity. Conserved motifs are indicated with horizontal bars on the top of the alignment. Predicted secondary structure is indicated below each block of sequences (orange: alpha-helix; blue: beta-sheet), except for A46 and N1, for which secondary structures of A52 (PDB:2VVW) and N1 (PDB:2I39), respectively, are shown. Green arrowheads indicate N1 protein residues putatively involved in BH3 peptide binding [11]. (B) Structural distribution of conserved motifs. Conserved residues in the multiple sequence alignment were mapped on the N1 structure (PDB:2I39). Secondary structure elements are depicted in yellow, except conserved residues, in orange. Side chains are coloured in red. Surface is shown in light grey. Structures were rendered with UCSF Chimera [60].

In order to find remote homologues of the proteins belonging to Pox_A46 family, we used sensitive Hidden Markov Models (HMM) profile-based searches through HHpred, a sequence homology search method based on HMM profile vs. profile comparisons [19]. A Pox_A46 family multiple sequence alignment from Pfam database was used as input to run HHpred against a database of all Pfam HMM profiles. The results confirmed the relationship between the Pox_A46 and Orthopox_N1 families (97.6% probability, e-value 3.4E-06), but also revealed the homology existing between the A46 family and two other families of poxvirus proteins: Pox_N2L (98.8% probability, e-value 1.6E-10) and Orthopox_C1 (72.5% probability, e-value 0.026). A similar search, started with the multiple sequence alignment of Pox_N2L family extracted from Pfam database, detected the Pox_A46 (99.9% probability, e-value 2.5E-25), Orthopox_C1 (97% probability, e-value 2.8E-06) and Orthopox_N1 families (74.5% probability, e-value 0.4). To detect every protein sequence related to these families, an iterative HMM search was started with the Pox_A46 HMM profile from Pfam database against a poxvirus protein sequence database. This search detected with significant e-values not only sequences containing the Pox_A46 domain, but also proteins belonging to other three Pfam families: Orthopox_N1, Pox_N2L and Orthopox_C1 (Additional File 1). Thus the sequence relationships among the four families were confirmed and all sequences belonging to any of them were collected. A multiple sequence alignment (Figure 1A) revealed that despite their size heterogeneity all these proteins contain a common conserved region of 110-140 residues at their C-terminal ends, leaving N-terminal ends of diverse lengths outside this region. For instance, in N1 (VACV-WR_028) the conserved region spans its whole length, while A46 (VACV-WR_172) has almost 90 extra N-terminal amino acids. A single HMM profile was built from the common conserved region of all these sequences and was used to refine the search. A HMMer search with this profile vs. UniProt database [20] found all and only the previously collected sequences. All the significant hits detected were poxvirus proteins. This result confirms the validity of the relationship among the four families (A46, N1, N2 and C1) and suggests that these four families should be merged into a single one.

Within this set of related poxvirus families three-dimensional structures are known for VACV proteins N1, A52, B15 and K7. They present a similar compact structure, formed by 6-7 alpha-helices, with outstanding similarity to the Bcl-2 family fold despite their lack of sequence homology with these cellular proteins. Homology at the sequence level with A46 and N1 families implies that members of the N2 and C1 families will probably adopt the same Bcl-2-like fold. Interestingly, the predicted secondary structure of the conserved region in N2 and C1 proteins is compatible with this fold (Figure 1A). To test the hypothesis that these proteins share the common domain of A46 and N1 families, multiple sequence alignments of N2 and C1 families were used to start HHpred searches against a sequence profile database derived from proteins with structures in the Protein Data Bank (PDB) [21]. A strong relationship was found between N2 and A52 structure (99.0% probability, e-value 1.3E-12). These results were supported by predicting the structure of this family with 3D-Jury [22], a fold recognition meta-server that obtains consensus predictions from different threading servers. In all cases the best hits were structures belonging to A46 and N1 families. Only in the case of C1 the results were not conclusive either with HHpred (42.5% probability, e-value 0.35) or with 3D-Jury (not shown). However, given that C1 sequence homology to N2 is evident from the HHpred searches, both families will probably share the Bcl-2-like common domain.

### Conserved residues in the common domain of the poxvirus protein families

Highly conserved amino acids of a multiple sequence alignment usually indicate that these residues are important for protein structure and/or function. In addition, amino acids that are conserved only in certain subfamilies are indicative of importance for specific functions carried out by these proteins subfamilies. A multiple sequence alignment of the common domain containing representative sequences of the four families (A46, N1, N2 and C1) was analyzed to get an insight of the conserved residues. The Proteinkeys web server [23] was used to find both conserved residues in all families and specific residues for individual families. Although the minimum sequence identity between the most divergent sequences of the four families can be as low as 15%, at least three conserved motifs could be distinguished in the multiple sequence alignment (Figure 1A): [LIVM]-x-x-Y- [IFL]-x- [WY]- [RS] in alpha-helix 1, G-x-x- [FY]-x-x- [LF]-x-x- [FYL]- [KD]-x-x-A in alpha-helix 2, and [IV]-G- [LF]-x- [ASG] in alpha-helix 5 (alpha-helices numbered according to N1). Since a common fold is assumed for all families, the sequence information was placed in the context of one of the known three-dimensional structures, that of N1 (PDB:2I39) (Figure 1B). Interestingly, alpha-helices 1, 2 and 5 are packed in close contact to one another in the common fold structure. Most of these conserved residues are hydrophobic and buried inside the protein core, so they are expected to have an essential role to preserve the domain structure stability. Because of their level of conservation and their position in the structure they might have been related to the pro-apoptotic peptide binding site.

Alpha-helix 1 forms part of the dimerization surface in N1, B15 and A52 proteins [11,12,14]. In the N1 homodimer residues Arg7 and Asp14 of alpha-helix 1 of different monomers form a potential salt bridge, contributing to dimer stability. This interaction is not found in A52 and B15 dimers as the relative orientation of monomers varies. Alpha-helix 2 is an amphipathic helix whose charged side is exposed and in the case of N1 contains several residues involved in BH3-peptide binding like Leu30, Glu32 and Leu33. The C-terminus half of alpha-helix 5 contains mostly hydrophobic residues and is buried in the protein core. One pair of amino acids identified by Proteinkeys as being conserved specifically in one subset of proteins is that of charged residues Arg12 and Asp31, which are located in conserved motifs in alpha-helices 1 and 2, respectively. These positions are highly correlated in the multiple sequence alignment, where both are present in a large subset of members of N1 and A46 families and completely absent in others. These amino acids join alpha-helices 1 and 2 through a potential salt bridge and probably contribute to the stability of BH3-peptide binding site structure. The same interaction is also conserved in K7 (Arg37 and Asp61) and A52 (Arg67 and Asp87) proteins. On the other hand there are a number of charged residues which are exposed on the surface of the proteins with known structure and seem relatively conserved in all families. For instance the pattern of charged residues alternating with hydrophobic residues in alpha-helix 2 is observed in N1, K7, B15 and A52 structures and it can be predicted in other proteins from their sequences. In N1 protein residues projecting outwards from alpha-helix 2 include Asp22, Lys25, Lys26 and Glu32, of which only the last one belongs to the ligand binding site [11]. Arg81 at the C-terminal end of alpha-helix 5 in N1 is exposed and charged residues at equivalent positions are conserved in A46 and N2 families. Conservation of these exposed residues may indicate a possible functionality, for instance an interaction with other proteins. Experimental data revealing detailed poxvirus-host protein interaction mechanisms are still scarce and more will be needed to confirm whether any of the conserved residues is functionally important.

### Evolutionary history of A46 and related families

**Figure 2:**
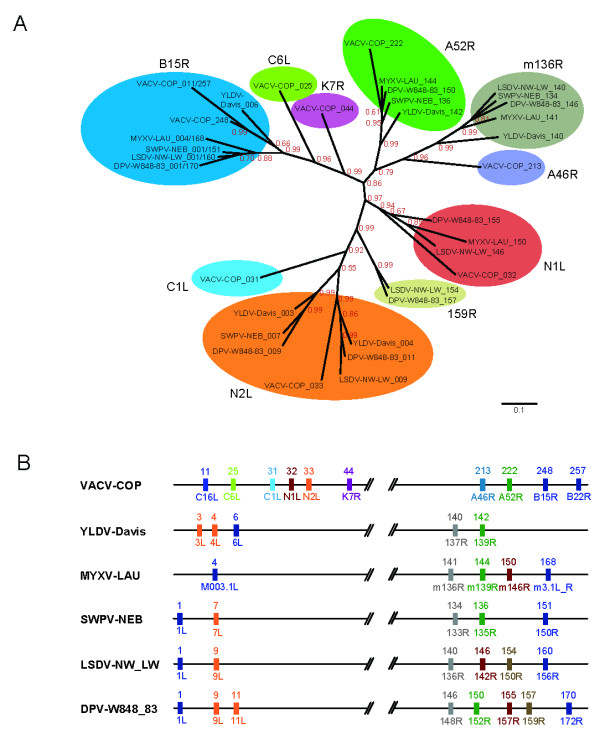
**Groups of orthologous genes in A46 and related families**. (A) Phylogenetic relationships among the orthologue groups obtained from A46, N1, N2 and C1 families. A Bayesian phylogenetic tree was constructed from a multiple sequence alignment of proteins encoded by genes in the ten orthologue groups. For simplicity only a representative species of every poxvirus genus, as depicted in (B), was selected. Posterior probabilities of every node are shown. (B) Virus genomes representing genera *Orthopoxvirus* (VACV-COP), *Leporipoxvirus *(MYXV-LAU), *Capripoxvirus *(LSDV-NW_LW), *Suipoxvirus *(SWPV-NEB), *Yatapoxvirus *(YLDV-Davis) and *Cervidpoxvirus *(DPV-W848_83) are depicted, indicating the relative genome positions of genes included in the orthologue groups. Species/strain names as in Figure 1A; VACV-COP, vaccinia virus strain Copenhagen. Numbers above every line represent the gene positions in the genome. Symbols below every line represent gene names. Genes drawn in the same colour belong to the same orthologue group.

In an attempt to reconstruct the evolutionary history of the whole family first we built its complete phyletic pattern, meaning by that the distribution of the subfamilies or groups of orthologues that integrate the gene family across all species of chordopoxviruses. Our gene set was divided into ten orthologue groups (Figure 2A). These orthologue groups are exclusively present in a monophyletic group that includes the genus *Orthopoxvirus *and a clade comprising five other genera (*Yata*-, *Capri*-, *Sui*-, *Lepori*- and *Cervidpoxvirus*), named Clade II by convention [24]. We could not find any remote homologue of this gene family in the remaining taxonomic groups of the poxvirus phylogeny. The distribution and number of genes of every orthologue group varies among different species (Figure 2B and Additional File 2), although they are always restricted to both terminal genome regions, where genes involved in virus-host interaction are usually located in poxvirus genomes [25,26]. Eight of the orthologue groups can be found in orthopoxvirus genomes: N1L, N2L, A52R and B15R can also be found in the Clade II species, whereas C6L, C1L, K7R and A46R are unique to orthopoxviruses. On the other hand two subfamilies are absent in this genus: those of orthologous genes to myxoma virus m136R and deerpoxvirus 159R, respectively.

**Figure 3:**
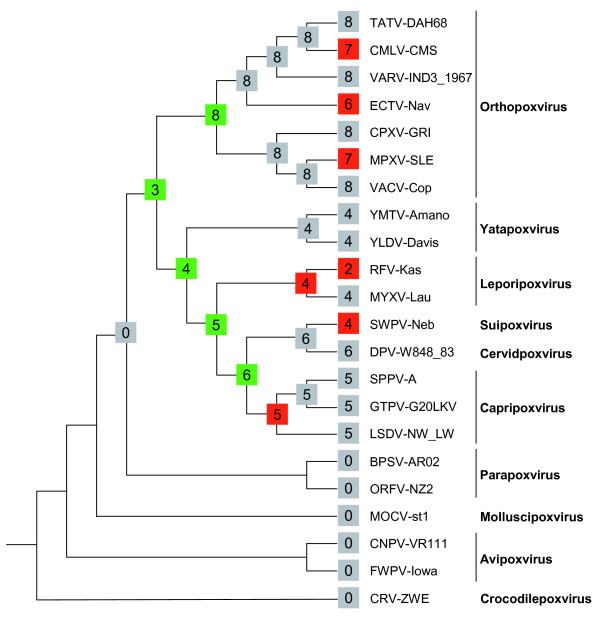
**Reconstruction of ancestral gene repertoires in the evolutionary history of A46 and related families**. The number in every node represents the inferred or real number of groups of orthologues present in each genome. This number was inferred for ancestral species by the maximum likelihood method implemented in the Count program [30]. The background colour of the number indicates the kind of variation in the gene content since the preceding node: green for nodes with a net gene gain, red for nodes with a net gene loss, and grey if the gene content remained unchanged. The tree contains a representative strain for every species of the subfamily *Chordopoxvirinae *with a completely sequenced genome and is based on a maximum likelihood phylogenetic tree (Additional File 3). Species/strain names as in Figures 1 and 2; TATV-DAH68, Taterapox virus strain Dahomey 1968; CMLV-CMS, Camelpox virus strain CMS; VARV-IND3_1967, Variola virus strain India 3 Major 1967; CPXV-GRI, Cowpox virus strain GRI-90; MPXV-SLE, Monkeypox virus strain Sierra Leone; YMTV-Amano, Yaba monkey tumor virus strain Amano; RFV-Kas, Rabbit fibroma virus strain Kasza; SPPV-A, Sheeppox virus strain A; GTPV-G20LKV, Goatpox virus strain G20-LKV; BPSV-AR02, Bovine papular stomatitis virus strain BV-AR02; ORFV-NZ2, Orf virus strain NZ2; MOCV-st1, Molluscum contagiosum virus strain subtype 1; CNPV-VR111, Canarypox virus strain ATCC VR111; FWPV-Iowa, Fowlpox virus strain Iowa; CRV-ZWE, Crocodilepox virus strain Zimbabwe.

The information provided by the phyletic pattern was superimposed on a consensus phylogenetic tree built from several single-copy conserved genes in all poxviruses. The topology of this tree was similar to other poxvirus phylogenies [27,28]. The family gene content evolution across the poxvirus phylogeny was reconstructed using the maximum likelihood method of Miklos and Csuros [29] implemented in the program Count [30]. This method allows inferring the genome sizes and gene repertoires of ancestral viruses, along with gene gain and loss events. The reconstruction of the evolutionary history of the family (Figure 3) suggests that the common ancestor of orthopoxviruses and the Clade II would have contained three genes of this family. Which orthologue group it could have belonged to cannot be deduced since probabilities are low for all of them (p < 0.5). As a comparison, reconstruction by parsimony suggests that this ancestor would have had four subfamilies (N1L, N2L, A52R and B15R). Less controversy exists between both methods for more recent ancestors. The common ancestor to all orthopoxviruses would have contained eight genes, what implies five gene gain events according to the maximum likelihood method. In this occasion the gene content of the ancestral virus is more evident as it most likely contained all eight orthologue groups present in practically every extant orthopoxvirus (with p = 1). In the branch leading to the Clade II its common ancestor would have possessed four genes belonging to this family, implying three gene gains over the preceding node. The four genes present in the ancestral genome were with p = 1 N2L, A52R, m136R and B15R. More recent evolutionary events include small gene gains and small gene losses in the branches leading to extant species. Altogether these data suggest that this gene family originated in the virus lineage leading to the common ancestor of orthopoxviruses and the Clade II, where between three and four gene gain events occurred. However it is unlikely that these gene gains occurred independently in a single ancestral virus. Furthermore, because of the evident sequence similarity among the putative genes in the ancestral virus genome, the most probable hypothesis would be that a Bcl-2 protein had been acquired from a eukaryotic host by the common ancestor of the subset of vertebrate poxviruses previously mentioned and probable events of gene duplication occurred within its genome before speciation proceeded. After the divergence of both poxviruses lineages new gene gain events increased the number of orthologue groups, probably because of the evolutionary advantage that these proteins conferred over the host organism in terms of regulation or suppression of antiviral immune response. However in more recent ancestors the overall number of subfamilies within poxvirus genomes appears to have stabilized. An explanation for this stabilization might be that the gene repertoire of this family was varied enough to accomplish its mission.

N1 is the only protein of this family with the same functionality as the putative Bcl-2 ancestor gene so far. While keeping the same basic tertiary structure these proteins evolved until they managed to bind a diverse range of cellular proteins involved in an important pathway in response to pathogen attacks. As yet the presence of only other three families of Bcl-2-like genes has been confirmed in poxviruses. They are vaccinia virus F1L [31] with orthologues in all orthopoxviruses, myxoma virus M11L [32,33] with orthologues in all genera of the Clade II, and fowlpox virus FPV039 [34] with orthologues in avipoxviruses. These are apparently single-copy genes and have no sequence similarity with the A46 and related Bcl-2-like families. Furthermore they lack sequence homology among them and only the avipoxvirus protein displays some sequence similarity with cellular Bcl-2 proteins. Very interestingly, these three families carry out the same function, apoptosis inhibition by binding pro-apoptotic BH3 peptides, but do not coincide in any poxvirus genome. Whether the origin of every poxvirus Bcl-2-like protein is independent or they arose from a gene present in a common ancestor of chordopoxviruses and any sequence relationship was lost during successive speciation events is undetermined. Nevertheless it is tempting to consider that the presence of other Bcl-2-like apoptosis inhibitors in poxvirus genomes offered the A46 and related families the opportunity to freely evolve.

### Functional considerations of the four protein families

**Figure 4:**
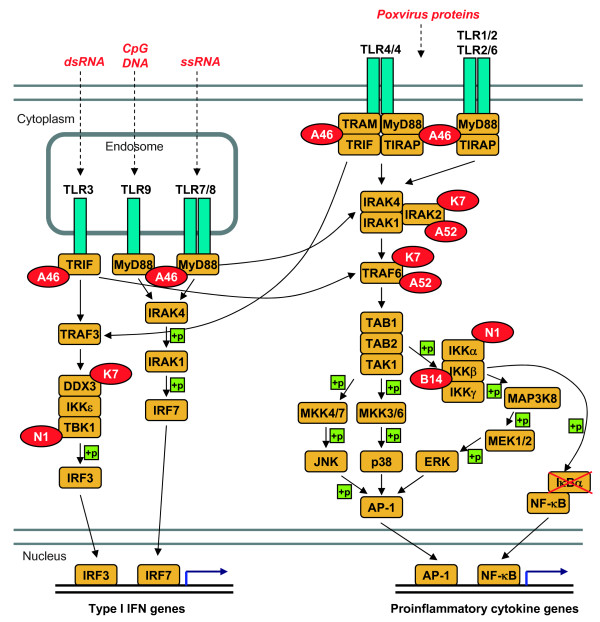
**Inhibition of host signalling pathways by VACV members of A46 and related families**. TLRs are distributed in the plasma membrane and endosomes. When a pathogen is recognized by a TLR adaptor proteins are recruited which transmit the signal further downstream until specific transcription factors are activated and enhance the expression of genes encoding type I IFNs and pro-inflammatory cytokines. VACV proteins belonging to A46 and N1 families interfere with the TLR signalling pathway at different levels. A46 targets all known adaptor proteins: MyD88, MAL (TIRAP), TRIF and TRAM. A52 targets IRAK2 and TRAF6, intermediary between adaptors and transcription factors. K7 inhibits IRAK2, TRAF6 and also DDX3, which is part of the complex that activates transcription factor IRF3. B15 targets the IKK complex by avoiding IKKbeta phosphorylation, what eventually causes the inhibition of NF-kappaB. N1 associates with several components of the IKK complex and with TBK1, inhibiting NF-kappaB and IRF3 activation, respectively.

The common structural core and the sequence homology to N1 might suggest that some of the other proteins belonging to A46, N2 and C1 families could be involved in an anti-apoptotic role as N1. However this functionality has yet to be proven. On the contrary, it has been discarded for A52 and B15 [14] and probably for K7 [16]. However, the proteins A46, A52, B15, K7 and N1 target diverse host participants of the TLR signalling pathway (Figure 4) that are apparently unrelated among them, suggesting that the mechanisms of action of these poxvirus proteins are heterogeneous. We describe below the information available thus far on A46, N1, N2 and C1 families regarding the functional characteristics of these proteins, which might help to infer the molecular mechanism of these functionalities and find whether these functions can be transferred to other proteins in these families.

N1 is the only of these families with an experimentally confirmed anti-apoptotic role. The N1 binding site to BH3 peptides consists basically of a hydrophobic groove flanked by charged residues [11]. Functional N1 residues are scarcely conserved in the rest of related families (Figure 1A). However, among the set of N1 residues which putatively interact with BH3 peptides, there are three residues (Ile75, Leu30 and Glu32) which belong to conserved motifs in alpha-helices 2 and 5. Proteins A52 and B15 do not inhibit staurosporine-induced apoptosis and this might be explained because in their surfaces the BH3-peptide binding groove would be blocked due to the greater length of alpha-helix 2, about one turn longer in comparison with that of N1 protein [14]. Alpha-helix 2 in N1 has 12 residues while in A52, B15 and K7 it comprises 17 residues. In most members of the families A46, N2 and C1, the length of alpha-helix 2 can be predicted because two conserved Gly residues usually delimit it, and in all cases it would have approximately the same length as in A52. Thus none of these proteins would be expected to have anti-apoptotic properties like N1, although experiments should be performed to confirm this hypothesis.

VACV A46 inhibits TLR signalling pathway by binding to MyD88 and TRIF adaptors, a TIR-like domain being likely responsible for these interactions. This TIR-like domain has not yet been found in other VACV proteins or other poxvirus proteins apart from close A46 homologues in orthopoxviruses. Three conserved sequence motifs of TIR domains were described along the A46 protein sequence [4,5]: one in its unique N-terminus and the other two in the alpha-helices 1 and 7 of the common domain with N1. Despite the sequence similarity in these motifs the overall predicted structure of A46 protein is not coincident with that of TIR domains, which in the case of TLR1 and TLR2 contain a central five-stranded parallel beta-sheet surrounded by five alpha-helices on both sides [35]. In fact we could not find any relationship of A46 or any other VACV protein with TIR domains by using tools for remote sequence homology search or fold recognition (data not shown). This seems to discard the straightforward explanation that A46 would have acquired its unique role by grabbing a functional TIR domain from a host cell genome. In fact, if A46 had really evolved from a remote Bcl-2-like ancestor and not from a TLR-like ancestor the origin of the TIR conserved motifs might have probably been due to mutations which constituted an evolutionary advantage for viruses containing this gene.

A52 inhibits TLR-dependent NF-kappaB activation by binding to both TRAF6 and IRAK2 [4,6]. Experiments with different mutant proteins have produced some data about A52 interaction with these host proteins at the molecular level. A deletion mutant including its N-terminal 144 residues was sufficient for inhibiting NF-kappaB activation and was able to interact with IRAK2 but not with TRAF6 [6], although it is not clear whether TRAF6 interacts with the A52 C-terminus. Moreover the N-terminal 36 residues of A52 were not required to inhibit IL-1alpha-induced NF-kappaB activation [14]. A small peptide from VACV A52 has been shown to mimic the function of the whole protein as it avoids TLR-dependent cytokine secretion [36]. Recent experiments demonstrating that A52 inhibits NF-kappaB activation by several TLRs only through its interaction with IRAK-2 but not TRAF6 [37] support the hypothesis that this peptide acts on IRAK-2. The sequence corresponding to the peptide is moderately conserved among A52 orthologues and poorly conserved among other related poxvirus proteins. On the other hand we could not find in A52 sequence a canonical TRAF6-binding motif, P-x-E-x-x-(acidic/aromatic), that was identified in several TRAF6 cellular interaction partners [38]. This suggests that A52 must bind TRAF6 through a different mechanism.

The crystal structure of K7 in complex with a 20 amino-acid DDX3 peptide has determined the precise details of their interaction [17]. DDX3 binds to a deep hydrophobic pocket in a negatively charged face of K7 delimited by its N-terminus, alpha-helix 1 and a non-helical segment equivalent to alpha-helix 6 in Bcl-2-like proteins. Interestingly, this region corresponds to the dimerization interface in A52, which differentiates from K7 in that it cannot bind DDX3. Like A52, K7 binds the TRAF domain of TRAF6 [15] but our search did not find a canonical TRAF6-binding motif in its sequence.

It is striking how proteins of these families evolved from a common Bcl-2-like domain with anti-apoptotic role to perform diverse functions always related with the inhibition of the host immune response, more specifically the TLR signalling pathway, but at different levels and using different mechanisms. These poxvirus proteins probably act at the level of subtle protein interaction to sequester a target protein or impede a complex formation, but their mechanisms of action are mostly unknown. Although the structures of some of these proteins have been elucidated, as yet only one of them represents a complex with a host target peptide, what still hinders the prediction of possible functions for other members of these families.

Experimental data are scarce or even absent for VACV proteins C1, C6, N2 and C16/B22. C6 protein has been found in a very low proportion in vaccinia virus IMV particles [39], as is the case of A46. One possible reason for their presence in the virion could be that they are necessary for the viral cycle early after virus entry. On the other hand a VACV attenuated strain with a C6L gene deletion has shown an enhanced immune response *in vivo *(manuscript in preparation), indicating that this protein may also be involved in the regulation of the host immune response. An early study revealed N2 location in the host cell nucleus during virus replication and discovered that a single nucleotide substitution in the 5'-UTR of N2L gene was responsible for an alpha-amanitin-resistant phenotype [40]. This data could suggest a possible function of N2 in transcription, although this hypothesis has not been confirmed yet. An experiment performed to determine interactions between VACV and host cell proteins revealed three possible interacting partners for C6 and other three for N2, as determined by yeast two-hybrid and validated by pull-down [41]. However none of them seems to be directly related with the host immune response. One of the C6 binding partners was programmed cell death 6 interacting protein (PDCD6IP/ALIX), which has been involved in apoptosis regulation, cytokinesis and HIV-1 budding. VACV C6 also interacted with keratin 4 (KRT4) and troponin I, skeletal, fast (TNNI2). In the same experiment three possible binding partners were described for N2: karyopherin alpha 2 (KPNA2), that may be involved in nuclear transport of proteins, phospholipid scramblase 4 (PLSCR4), that participates in the regulation of the movements of phospholipids in membranes, and valosin containing protein p97/p47 complex interacting protein 1 (VCPIP1), a deubiquitinating enzyme required for Golgi and ER assembly. These interaction data can help to uncover possible roles of C6 and N2, although they must be taken cautiously until more specific experiments are performed. To our knowledge, no experimental data have been published yet about VACV proteins C1 or C16/B22.

Recent studies on vaccinia virus transcription revealed the existence of an immediate-early class of genes [42]. This class includes five genes of this family (A52R, B15R, C6L, K7R and N2L), while other five (A46R, N1L, C1L and C16L/B22R) belong to the early class. An immediate-early or early expression pattern can be characteristic of proteins involved in immune response evasion. Thus, those data agree with the known functions of A46, A52, B15, K7 and N1, and may support a possible role in immune response evasion of the members of these families with still unknown function.

The above findings have implications in the use of poxviruses as vaccines, in particular vaccinia virus attenuated strains MVA [43,44] and NYVAC [45] that have been studied extensively [46]. In comparison with strain WR, MVA lacks A52R and C1L genes while NYVAC lacks C6L, N1L, N2L and C1L genes. However MVA contains one (MVA189R) and NYVAC contains two (C16L/B22R) additional genes with similarity to B15R which are not present in strain WR. A major difference in behaviour between these attenuated strains is that NYVAC provokes greater cytopathic effect, phosphorylation of EIF2-alpha and apoptosis in infected cells [47]. C6L, N2L and N1L are among the genes present in MVA and absent in NYVAC and thus could explain this behaviour.

## Conclusions

We have described the sequence relationship among four families of poxvirus proteins, A46, N1, N2 and C1, which share a common domain with a Bcl-2-like fold, and proposed their integration into a single family. The phylogenetic distribution and reconstruction of the evolutionary history of this family indicate that it originated in the common ancestor of orthopoxviruses and a clade formed by five other poxvirus genera. After initial increases in the family gene content in the most ancestral viruses a balance between gene gains and losses appears to have stabilized the number of family members in extant poxviruses. Their roles determined so far indicate that these proteins have specialized in regulating the host immune response, clearly suggesting that similar functions should be researched for other members of this family with still undefined function, like N2, C1, C6 and C16/B22. The diversity of host targets and the lack of precise data about what residues are involved in poxvirus-host protein interactions hamper the prediction of new targets for these families. Nevertheless, based on secondary structure predictions, our analysis foresees that practically all members of this family will be unable to bind pro-apoptotic peptides and inhibit apoptosis as N1 does. This study highlights the relevance of poxvirus protein families in innate immune sensing and suggests, from a point of view of the application of attenuated poxviruses as vaccines, that to avoid redundancy in related functions, gene deletions of entire families should be considered when recombinant vectors are developed with improved immune capacity.

## Methods

### Sequence homology analysis

Poxvirus protein sequences were obtained from the Poxvirus Bioinformatics Resource Center database [48,49].

Multiple sequence alignments of families were retrieved from Pfam database version 23 [18] when indicated. A global sequence alignment was obtained with MAFFT [50] using the L-INS-i mode with default parameters and including three-dimensional structures to guide the alignment. The alignment was then manually adjusted.

Profile versus profile searches were performed with HHpred [19] in the global alignment mode and scoring secondary structure. Searches were carried out against Pfam-A_23 and PDB70 HMM profile databases available in the same web server.

Iterative searches with HMMer [51], a method based on HMM profile vs. sequence comparisons, were performed as follows. A single search was started with a HMM profile against a database of poxvirus protein sequences. All hit sequences below a threshold e-value of 0.01 were automatically aligned and from the alignment a new HMM profile was built which was used to start a new search. This was performed several rounds until the search reached the convergence, i.e. no new sequences were added.

Secondary structure predictions were performed with PsiPred [52] starting from multiple sequence alignments of single families.

### Phylogenetic analyses

The Bayesian phylogenetic tree of representative proteins of orthologue groups was obtained by running MrBayes v3.1.12 [53,54] for 100000 generations in two rounds of two chains each through the Phylemon web server [55]. Trees were visualized with Phylodendron [56].

For the poxvirus phylogenetic tree concatenated alignments of proteins encoded by five single-copy conserved poxvirus genes (E9L, J3R, J6R, H6R and D5R) from every chordopoxvirus species with at least one fully sequenced genome were used. An entomopoxvirus species was used as an outgroup to root the tree. The maximum likelihood phylogenetic tree was built with PhyML v3.0 [57] with the LG substitution model, four substitution rate categories, estimated proportion of invariable sites and branch support estimated by non-parametric bootstrap analysis with 100 replicates.

### Reconstruction of the family gene content evolution

Groups of orthologous proteins were detected by using the bidirectional best hit method. Starting with a dataset containing all poxvirus sequences, a BlastP [58] search was performed with every sequence within or with homology to the A46 family against the whole dataset. Two proteins belonging to different species were considered orthologues if each was the best hit of the other in their respective species. The orthologue groups obtained were contrasted with the Poxvirus Orthologous Clusters [59] from the Poxvirus Bioinformatics Resource Center database. For simplicity, several paralogues were included in orthologue groups in the cases of orthopoxvirus proteins in the B15R group and Clade II proteins in the N2L group.

The gene content evolution was reconstructed with Count [30]. Input data comprised a table with the distribution of the groups of orthologous genes across the chordopoxvirus genomes (Additional File 2) and the poxvirus phylogenetic tree (Additional File 3). The ancestral reconstruction by likelihood maximization based on a phylogenetic birth-and-death model was chosen [29]. Rate optimization was performed using a gain-loss-duplication model with a Poisson family size distribution at the root. Family sizes and lineage-specific events (gains, losses, expansions and contractions) were computed using posterior probabilities in the optimized gain-loss-duplication model.

## Competing interests

The authors declare that they have no competing interests.

## Authors' contributions

JMG carried out the bioinformatics analyses, participated in the design of the study and drafted the manuscript. ME conceived the study, participated in its design and helped to draft the manuscript. All authors read and approved the final manuscript.

## Supplementary Material

Additional file 1 **Poxvirus protein sequences detected by an iterative HMM search**. Poxvirus protein sequences detected with an e-value < 1 in the final round after an iterative HMM search started with the Pox_A46 HMM profile from Pfam database against a poxvirus protein sequence database from the Poxvirus Bioinformatics Resource Center http://www.poxvirus.org. Click here for file

Additional file 2 **Distribution of orthologue groups across poxvirus genomes**. Table that displays the number of genes of every orthologue group (rows) across every poxvirus species (columns). Click here for file

Additional file 3 **Maximum likelihood phylogenetic tree of poxvirus species (Newick format)**. Maximum likelihood phylogenetic tree built from concatenated alignments of sequences of proteins encoded by five single-copy conserved poxvirus genes (E9L, J3R, J6R, H6R and D5R) from every chordopoxvirus species with at least one fully sequenced genome. Protein sequences from an entomopoxvirus (AMEV-Moyer) were included to root the tree. Click here for file
